# New Baitouweng decoction combined with fecal microbiota transplantation alleviates DSS-induced colitis in rats by regulating gut microbiota metabolic homeostasis and the STAT3/NF-κB signaling pathway

**DOI:** 10.1186/s12906-022-03766-z

**Published:** 2022-11-24

**Authors:** Xin Gu, Zhiwei Miao, Yantian Wang, Yue Yang, Tongtong Yang, Yi Xu

**Affiliations:** 1 Department of Gastroenterology, Affiliated Hospital of Nanjing University of Chinese Medicine, Jiangsu Province Hospital of Chinese Medicine, Nanjing, China; 2 Department of Gastroenterology, Zhangjiagang TCM Hospital Affiliated to Nanjing University of Chinese Medicine, Zhangjiagang, China

**Keywords:** Ulcerative colitis, New Baitouweng decoction, Fecal microbiota transplantation, Gut microbiota, STAT3/NF-κB signaling pathway

## Abstract

**Aim of the study:**

We aimed to elucidate the synergistic effect and potential mechanism of New Baitouweng Decoction (NBD) combined with fecal microbiota transplantation (FMT) in rats with DSS-induced ulcerative colitis (UC).

**Materials and methods:**

Colitis was induced by 5% (w/v) dextran sulfate sodium (DSS) in drinking water for 7 days. NBD or NBD combined with FMT were administered to the colitis rats. Body weight and disease activity index were measured, and the colon histological change was imaged to further examine the efficacy of NBD and FMT. The specific effects of NBD on STAT3/NF-κB signaling pathway and gut microbiota in rats with UC were also investigated.

**Results:**

The efficacy of NBD in combination with FMT was demonstrated by the lower disease activity index scores; increased tight junction proteins expression; and a lower expression of macrophage marker (F4/80) in colon tissues. NBD combined with FMT elevated the concentrations of short-chain fatty acids and inhibited activation of the JAK2/STAT3/NF-κB related proteins. Furthermore, 16SrDNA sequencing indicated that the gut microbiota in rats with UC was perturbed, in contrast to that in healthy rats. After treatment with NBD and FMT, the diversity and abundance of intestinal flora showed clear improvements. Spearman correlation analysis indicated a strong correlation between specific microbiota and fecal concentrations of acetate, propionate and butyrate.

**Conclusions:**

The protective mechanism of NBD combined with FMT may be linked to regulation NF-κB/STAT3 and restoration of the intestinal flora.

**Supplementary Information:**

The online version contains supplementary material available at 10.1186/s12906-022-03766-z.

## Background

Ulcerative colitis (UC), a highly prevalent chronic disease, has a complex and unclear pathogenesis, but is considered to be associated with the interaction of genetic factors, the external environment, autoimmune dysfunction and imbalances in the intestinal flora [1]. Monotherapies with aminosalicylates, corticosteroids, immunosuppressive drugs and biological agents have poor efficacy and cause many adverse effects [2]. For aggressive UC, combination therapy at an early stage is the most appropriate treatment [3]. Combination treatment decreases inflammation and regulates the intestinal flora, thus significantly improving ulcerative symptoms [4, 5]. Therefore, combination treatment may be better than monotherapy.

Recent studies have demonstrated that imbalances in the intestinal microflora are a paramount factor in the initiation and progression of UC. Imbalances in the gut microbiota alter microbial diversity, and consequently disrupt symbiosis of microbiota and the host [6]. In addition, the intestinal microbiota derived metabolites could trigger mucosal immune response in colon, thereby disrupting the immune function of the intestinal mucosa and triggering UC [7]. Recently, fecal microbiota transplantation (FMT) has become a pleasurable therapeutic approach for UC. FMT could induce intestinal microecological changes in patients in whom biological and immunosuppressive agents are ineffective, thus enhancing the therapeutic effects of other drugs. A clinical study has indicated that the clinical efficacy of messalamine combined with probiotics in patients with mild-to-moderate UC has greater efficacy than messalamine alone [8]. The improvements in the gut microbiota are believed to affect the regulation of nuclear factor-kappa B (NF-κB), a gene transcriptional regulator crucial in the inflammatory process [9]. When inhibitor of NF-κB (IκB)α undergoes phosphorylation and degradation, the downstream NF-κB signaling pathway is activated, thus initiating target gene transcription and mRNA formation. In addition, the binding of cytokines to the transmembrane receptor induces the phosphorylation of the Janus kinase (JAK), thus stimulating signal transduction and transcriptional activator 3 (STAT3). Activated STAT3 is involved in the gene transcription and protein expression of a variety of inflammatory factors, thereby promoting the formation and persistent exacerbation of inflammation [10]. Thus, regulating JAK2/STAT3/NF-κB signaling is an effective strategy to treat UC.

Traditional Chinese medicine has become a common alternative treatment for UC because of its flexibility and low toxic side effect. Baitouweng decoction containing *Pulsatilla chinensis* (Bunge) Regel (Baitouweng), *Phellodendron chinense* C. K. Schneid. (Huangbai), *Fraxinus chinensis* Roxb. (Qinpi) and *Coptis chinensis* Franch. (Huanglian), a classical traditional Chinese medicine prescription, originated from “Shang Han Lun” written by Zhang Zhongjing [11]. Several studies have shown that Baitouweng decoction has a variety of effects including anti-diarrhoeal, anti-inflammatory and regulation of intestinal microbiota [12]. However, the development of UC is complex and related to depression and oxidative stress [13, 14]. Previous experiments have concluded that Baitouweng decoction is less than ideal for treating UC [11]. Therefore, based on clinical experience, we added several herbs to Baitouweng decoction to form New Baitouweng Decoction (NBD) for better treatment of UC. NBD has been demonstrated their efficacy in our previous clinical efficacy assessment [15]. Also, our previous study has indicated that a NBD-related herbal granule produced by the Affiliated Hospital of Nanjing University of Chinese Medicine improves experimental colitis by restoring dendritic cell-mediated Th17/Treg balance [16]. NBD, known as Qingchang Huashi Recipe, has been shown to inhibit the inflammatory response of HT-29 cells and macrophage chemotaxis and to reduce NF-κB activation [17]. In addition, both *Aucklandia lappa* and *Paeonia lactiflora* existing in NBD, were with anti-depressant and anti-inflammatory properties, which have been shown to have anti-ulcer effect [18, 19]. *Paeonia lactiflora Pall* and *Coptis chinensis* could alleviate UC through downregulating the NF-κB/STAT3 signaling pathway [20, 21].

We therefore hypothesized that NBD combined with FMT might improve UC through regulating immunity and the intestinal flora, and might have better effects than either treatment alone. We explored the effect of NBD administration in combination with FMT in a dextran sulfate sodium (DSS)-induced colitis model. Our study validated the possible mechanism of NBD in combination with FMT in DSS-induced UC by examining the JAK2/STAT3/NF-κB signaling pathway. Moreover, we used 16 SrDNA sequencing technology to investigate the structure of gut microbiota in colitis rats under combination therapy.

## Materials and methods

### Animals

Male Sprague–Dawley rats (weight, 180–220 g) were purchased from Qinglongshan Animal Breeding Farm, Jiangning District, Nanjing, China [license No: SYXK (Su) 2018–0049]. The rats were housed in an air-conditioned room (temperature 24–25 °C, humidity 60–65%) under a 12 h light/dark cycle. Animal experiments were approved by the Ethics Committee of Zhangjiagang TCM Hospital Affiliated with Nanjing University of Chinese Medicine. Ethics No.: AEWC-20201201.

### Preparation of NBD

The components of NBD were listed in Table 1. All crude medicines were purchased from the Affiliated Hospital of Nanjing University of Chinese Medicine and identified by Professor Cao Yuan. The above materials were boiled in water (1630 mL) for 1 h after soaking for 1 h. Dregs of the decoction were boiled in boiling water (1304 mL) for 1 h and filtered. The filtrate was combined and concentrated into 1 g crude drug/ml and then stored at 4 °C.

**Table 1:** The components of NBD

Chinese Name	Latin Name	Parts used in medicine	Proportion
Baitouweng	*Pulsatilla chinensis* (Bunge) Regel	Root	10
Huangbai	*Phellodendron chinense* C. K. Schneid	Cortex	10
Qinpi	*Fraxinus chinensis* Roxb.	Cortex	15
Huanglian	*Coptis chinensis* Franch.	Root	3
Baishao	*Paeonia lactiflora* Pall.	Root	15
Chishao	*Paeonia veitchii* Lynch	Root	15
Danggui	*Angelica sinensis* (Oliv.) Diels	Root	10
Mudanpi	*Paeonia suffruticosa* Andrews	Root cortex	10
Zicao	*Lithospermum erythrorhizon* Siebold & Zucc.	Root	15
Diyu	*Sanguisorba officinalis* L.	Root	15
Xianhecao	*Agrimonia pilosa* Ledeb.	Herba	30
Muxiang	*Aucklandia lappa* DC.	Root	10
Gancao	*Glycyrrhiza uralensis* Fisch.	Root and rhizome	5

### Analysis of components in NBD

Concentrated NBD extract was mixed with methanol and then centrifuged at *18,000* g for 10 min. The collected supernatant was subjected to LC-HR-MS analysis. The mobile phase consisted of 0.05% formic acid–water (phase A) and 0.05% formic acid–acetonitrile (phase B). The gradient elution program was as follows: 0–3.2 min, 10% B; 3.2–36 min, 10–90% B; 36–39.1 min, 90% B; 39.1–40 min,90–10% B; and 40–42.1 min, 10% B.

### Preparation of fecal microbiota transplantation

Fresh feces from healthy rats were sent to Nanjing Hilshou Biotechnology Co., Ltd. to make capsules. The capsules were 2.7 mm in diameter and dissolved only in the colon. Each capsule contained approximately 15 mg of extract with a bacterial content of 10^9^ cfu/g. Fecal bacteria capsules were cryopreserved at − 80 °C.

### Induction of the colitis model in rats and treatment with NBD and FMT

All rats were randomly divided into five groups: control group (Ctrl), DSS group (DSS), DSS + NBD group (NBD,17 g/kg), DSS + FMT group (FMT, 0.5 g/kg) and DSS + NBD + FMT group (NBD,17 g/kg; FMT, 0.5 g/kg). Except for the Ctrl group, which was given water, rats in the other four groups were given 5% dextran sulfate sodium (DSS) (Yeasen Biotech Co., Ltd., Shanghai, China) for 7 days to establish the UC model and then were then provided with water for another 3 days. The DSS + NBD group, DSS + FMT group and the combination group were treated with NBD, FMT and NBD combined with FMT from the second day of DSS intervention respectively. All rats were anesthetized by inhalation of 3% isoflurane (WANQING chemical Glass warey & Instrument Co., Ltd.) and sacrifced by cervical dislocation. After anesthetic euthanasia, the colons were removed, rinsed with phosphate buffered saline (PBS) and measured. The colons and feces were cryopreserved at − 80 °C until further analysis.

### Assessment of the disease activity index (DAI) score

During the experiment, daily body weight, stool consistency and the presence of occult blood in feces were documented to illustrate the overall status of the rats [22]. Specifically, five levels of weight loss were recorded, from 0 to 4, indicating no weight loss, < 5% loss, 5–10% loss, 10–20%, and > 20% loss, respectively. Diarrhea was graded on a scale of 0, 2 and 4, indicating normal stools, loose stools and watery diarrhea, respectively. Similarly, hematochezia was quantified with scores of 0, 2 and 4, representing no bleeding, slight bleeding and gross bleeding, respectively.

### Histology analysis

Colon sections (approximately 1 cm) were immediately fixed in 10% buffered formalin after being rinsed with ice-cold PBS. Then colon sections were subjected to hematoxylin and eosin (H&E) staining and then examined in a light microscopic to assess histological changes in pathological sections, and the Chiu standard grading was performed in Supplementary Table S1 [23].

### Quantitative real-time polymerase chain reaction (qRT-PCR)

Total RNA was extracted from the colon tissues with RNA isolater (Vazyme BioTech Co., Ltd., Nanjing, China) and then subjected to reverse transcription. The cDNA was then used to perform qPCR with ChamQ SYBR qPCR Master Mix (Vazyme BioTech Co., Ltd. in Nanjing, China). A CFX Connect RT-PCR Detection System (Bio-Rad, Hercules, CA, USA) was used to conduct qPCR. Gene expression analysis was performed by using the 2^−ΔΔCt^ method. Primer sequences are shown in Supplementary Table S2.

### Western blot analysis

Colonic tissues were completely homogenized with RIPA lysis buffer (Beyotime Biotech Co., Ltd., Shanghai, China). Next, the denaturated proteins were separated with SDS-polyacrylamide gel electrophoresis and electro-blotted onto a polyvinylidene fluoride (PVDF) membrane. The membranes were submerged for 2 h in blocking solution before incubation with the antibodies against glyceraldehyde-3-phosphate dehydrogenase (GAPDH) (60004–1-Ig, Proteintech); occludin (13409–1-AP, Proteintech); zona occludens-1 (ZO-1) (21773–1-AP, Proteintech); STAT3 (AP0366, Bioworld); phospho (p)-STAT3 (AP0248, Bioworld); NF-κB p65 (66535–1-Ig, Proteintech); phospho (p)-NF-κB p65 (#3033S, Cell Signaling Technology); JAK2 (ET1607–35, HUABIO); and phospho (p)-JAK2 (ET1607–34, HUABIO). Then, all PVDF membranes were incubated with secondary antibodies, bands were exposed with enhanced ECL solution (Yeasen Biotech Co., Ltd., Shanghai, China) and analyzed in Image J software (National Institutes of Health, USA, 1.8.0). (The blots cut prior to hybridization with antibodies were presented the cropped blots in the manuscript).

### Immunofluorescence analysis

First, colonic tissues dewaxed, rinsed and sectioned. The sections were incubated with various primary antibodies. After sections were incubated with secondary antibodies for 1 h, 4′6-diamidino-2-phenylindole (DAPI) staining solution (Beyotime Biotechnology, Shanghai, China) was used. Finally, confocal images were acquired with a LEICA STELLARIS confocal microscope (Leica Camera AG, Germany).

### Targeted short-chain fatty acid (SCFA) quantitative analysis

SCFA concentrations (acetate, propionate, butyrate, isobutyrate, valerate and isovalerate) in rat feces were determined by using previously reported methods [24]. Briefly, fecal samples (approximately 50 mg) were added proportionally to 0.5 ml 50% acetonitrile (10 μl solvent per 1 mg stool) and vortexed until fully mixed. The samples were centrifuged at *18,000* g at 4 °C for 5 min. The supernatant (30 μL) was combined with 180 mM 3-nitrophenylhydrazine in 50% acetonitrile (30 μL)and 100 mM EDC-6% pyridine solution (30 μL). The mixture was reacted at 40 °C and maintained for 30 min before centrifuged at *12,000* g at 4 °C for 10 min. Then, 50 μL supernatant collected was diluted to 100 μL with 50% aqueous acetonitrile for liquid chromatograph-mass spectrometer (LC-MS)/MS analysis.

### 16 SrDNA sequencing and sequencing data analysis

The DNA extracts of colon contents were detected and examined. Then DNA samples were sent to Majorbio Bio-Pharm Technology Co. Ltd. (Shanghai, China) for 16 SrDNA sequencing. The microbial diversity detection was performed on the V3-V4 hypervariable region of the 16SrDNA of the bacteria. The sequencing was performed with the PE300 sequencing strategy on the Illumina MiSeq platform (Illumina, San Diego, USA).

The SILVA (version 138) database was used to compare the bacterial diversity. Observed taxonomic unit (OTU) clustering of non-repeat sequences was performed with Uparse (version 7.0.1090) software, according to 97% similarity, and chimeras were removed. According to the silva138/16s_bacteria species classification database, OTU representative sequences of 97% similar levels were compared with the RDP classifier Bayesian algorithm. With the UniFrac algorithm, principal coordinate analysis (PCoA) was performed to compare the beta diversity of species community among samples. Classifications of bacteria among different groups at the phylum and genus levels were compared using the Kruskal-Wallis rank sum test. The dominant bacterial communities with statistical differences were analyzed using line discriminant analysis (LDA) effect size (LEfSe).

### Statistical analysis

All results are expressed as the mean and standard error of mean. One-way analysis of variance was used to analyze the differences between groups. *P*-values less than 0.05 were considered to indicate statistical significance, reported as follows: *P < 0.05, **P < 0.01 and ***P < 0.001.

## Results

### Identification of the main constituents of NBD

**Fig. 1:**
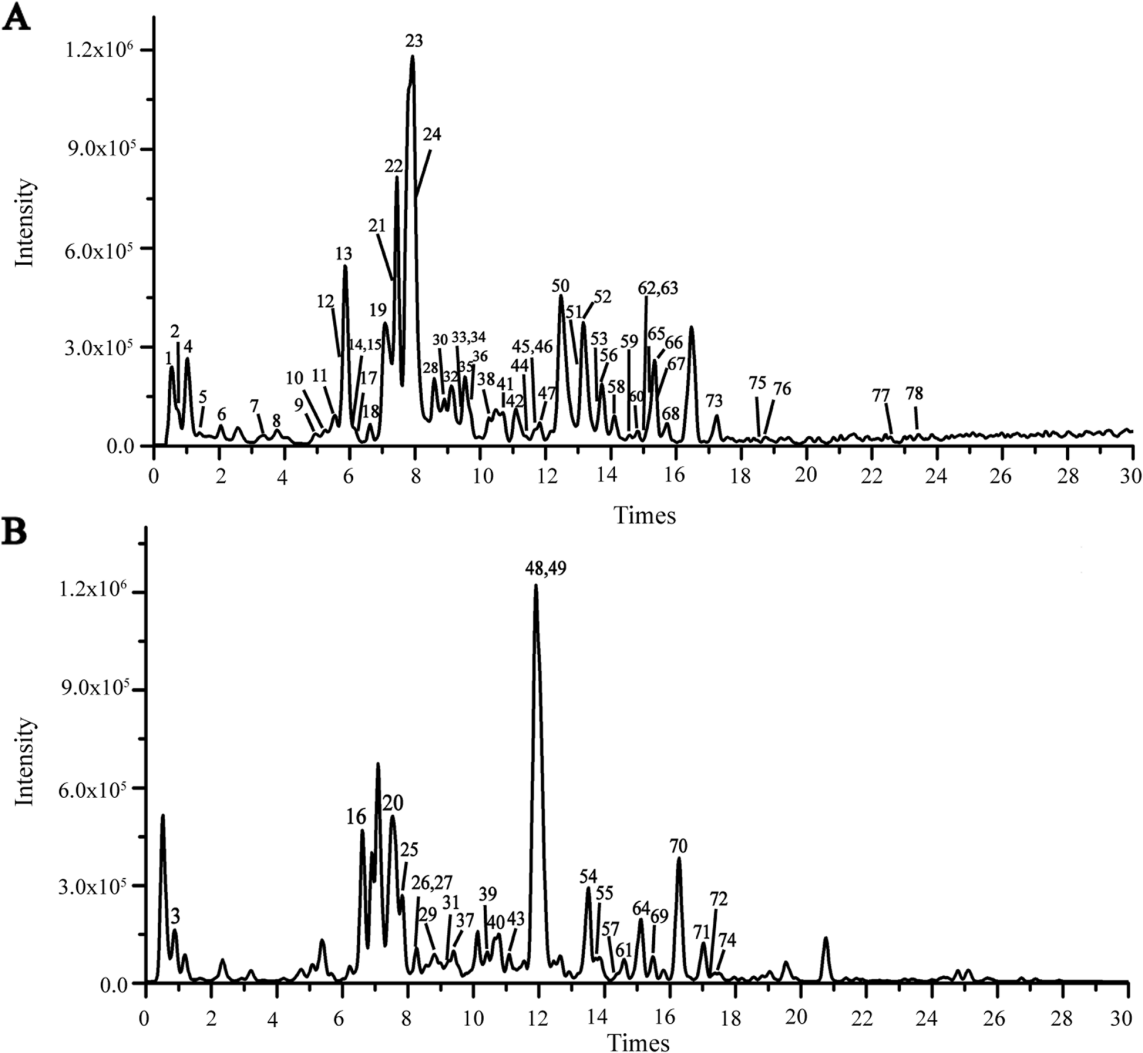
Base peak ion flow chromatogram of the major constituents of NBD. (**A**) Negative ion mode. (**B**) Positive ion mode

Figure 1 shows the total ion chromatography of NBD in positive and negative ion modes. A total of 78 components were identified from NBD, including 26 terpenoids, 10 flavonoids, 15 alkaloids, six coumarin components, 12 phenolic components, six organic acids, one naphthoquinone component and two other components. In addition, the identified main chemical components of NBD with analyzed retention times, precise molecular ion peaks and secondary mass spectrometry cleavage fragments are shown in Supplementary Table S3.

### NBD combined with FMT alleviated colonic injury in DSS-induced colitis in rats

**Fig. 2:**
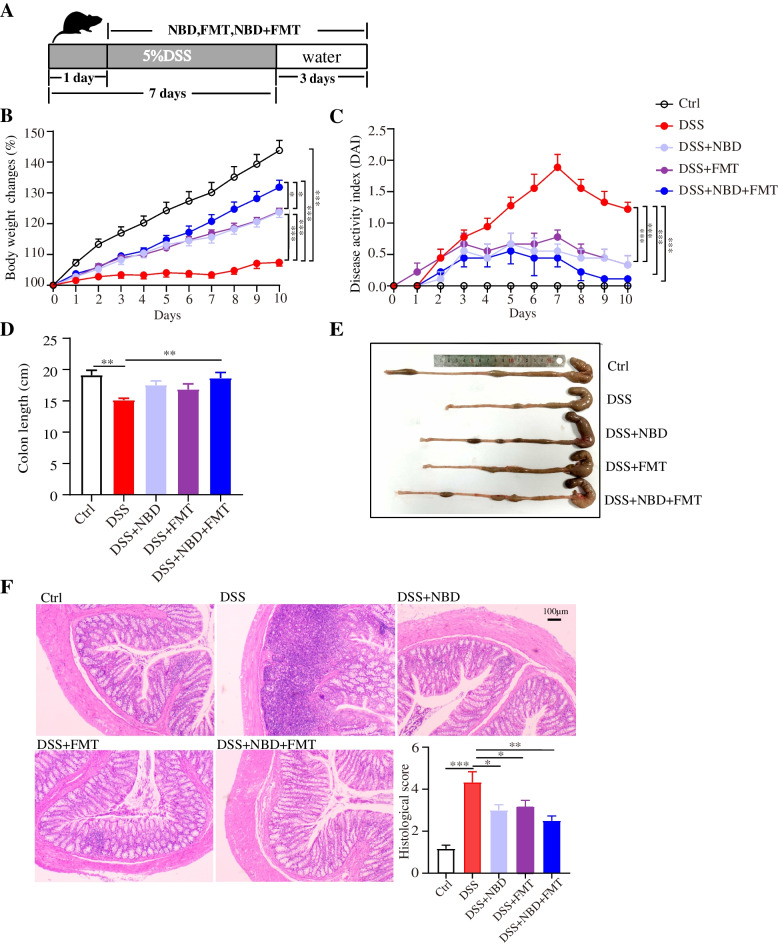
NBD combined with FMT attenuated DSS-stimulated UC. (**A**) Experimental schematic diagram, (**B**) body weight loss, (**C**) DAI score, (**D,E**) shortened colon length, (**F**) H&E staining and histological colitis score. Data are expressed as mean ± SEM, *P < 0.05, **P < 0.01, ***P < 0.001, *n* = 6

We evaluated the efficacy of three treatments in a rat model of colitis induced by 5% DSS (Fig. 2A). The body weights of the rats with UC were clearly lower than those of the rats in the Ctrl group (*P* < 0.001), whereas the body weights of the rats with NBD combined with FMT treatment were significantly closer to those of the rats in the Ctrl group (*P* < 0.001) (Fig. 2B). Obviously, the weight gain of rats in the combination treatment group was more significant than that in the NBD group and the FMT group (*P* < 0.05). Furthermore, the DAI score was also markedly decreased by treatment (*P* < 0.001) (Fig. 2C). The colon length in DSS-induced rats with UC was shorter than that in rats in the Ctrl group, but NBD combined with FMT prevented the DSS-induced colon shortening (*P* < 0.01) (Fig. 2D,E). Notably, three treatments were effective in alleviating the symptoms of colitis in rats. NBD combined with FMT showed more significant efficacy. Although the pathological staining results of the colon tissues indicated that rats with colitis in the DSS group had symptoms of disruption of epithelial tissues, mucosal layer incompleteness and massive infiltration of inflammatory cells; all these symptoms were markedly ameliorated by NBD and FMT treatment (Fig. 2F). Together, our data indicated that NBD combined with FMT significantly alleviated DSS-stimulated colitis.

### NBD combined with FMT up-regulated tight junction protein expression

**Fig. 3:**
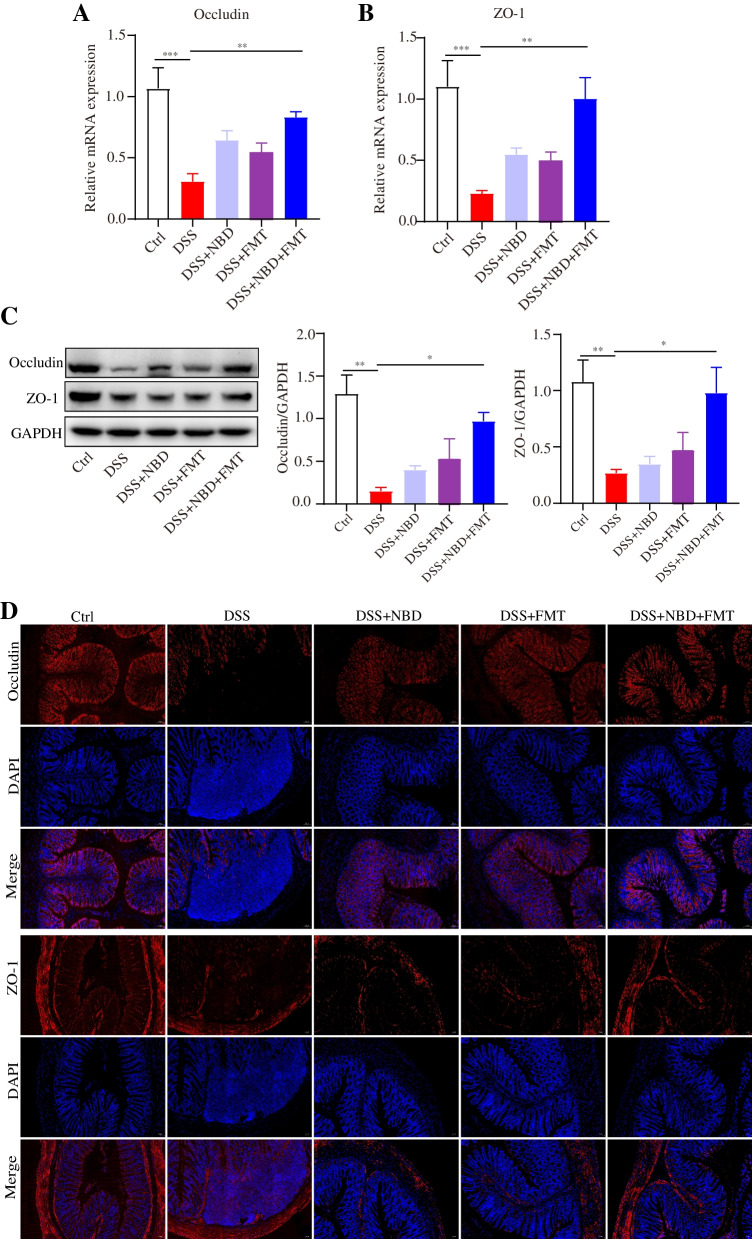
NBD combined with FMT up-regulated tight junction protein expression in colitic rats. (**A,B**) The expression of occludin (**A**) and ZO-1 (**B**) mRNA was down-regulated in the DSS group but up-regulated by NBD combined with FMT, *n* = 6. (**C**) Western blotting indicated that NBD combined with FMT elevated the protein expression of occludin and ZO-1 beyond that in DSS-induced rats with UC, *n* = 3. (**D**) Representative images of DAPI (blue), occludin and ZO-1 immunostaining (red) in colonic sections (× 200 magnification). Data are expressed as mean ± SEM, *P < 0.05, **P < 0.01, ***P < 0.001

We measured the representative tight junction proteins and found that the mRNA expression of ZO-1 and occludin were diminished in the DSS group, but combination treatment reversed this response (*P* < 0.001 or *P* < 0.01) (Fig. 3A,B). Additionally, the protein expression levels of occludin and ZO-1 decreased in the colon of UC rats but were upregulated by NBD combined with FMT (*P* < 0.01 or *P* < 0.05) (Fig. 3C). Interestingly, no significant differences were observed between the NBD group or the FMT group versus the DSS group (*P*>0.05). Combination treatment upregulated tight junction protein significantly more than NBD and FMT treatment, but there was no significant difference (*P*>0.05). Moreover, immunofluorescence staining indicated that DSS-induced colitis downregulated the protein expression of ZO-1 and occludin in the colon (*P* < 0.01 or *P* < 0.05) (Fig. 3D). However, NBD combined with FMT restored the expression levels of these proteins.

### NBD combined with FMT inhibited the inflammatory cytokines in DSS-stimulated colitis

**Fig. 4:**
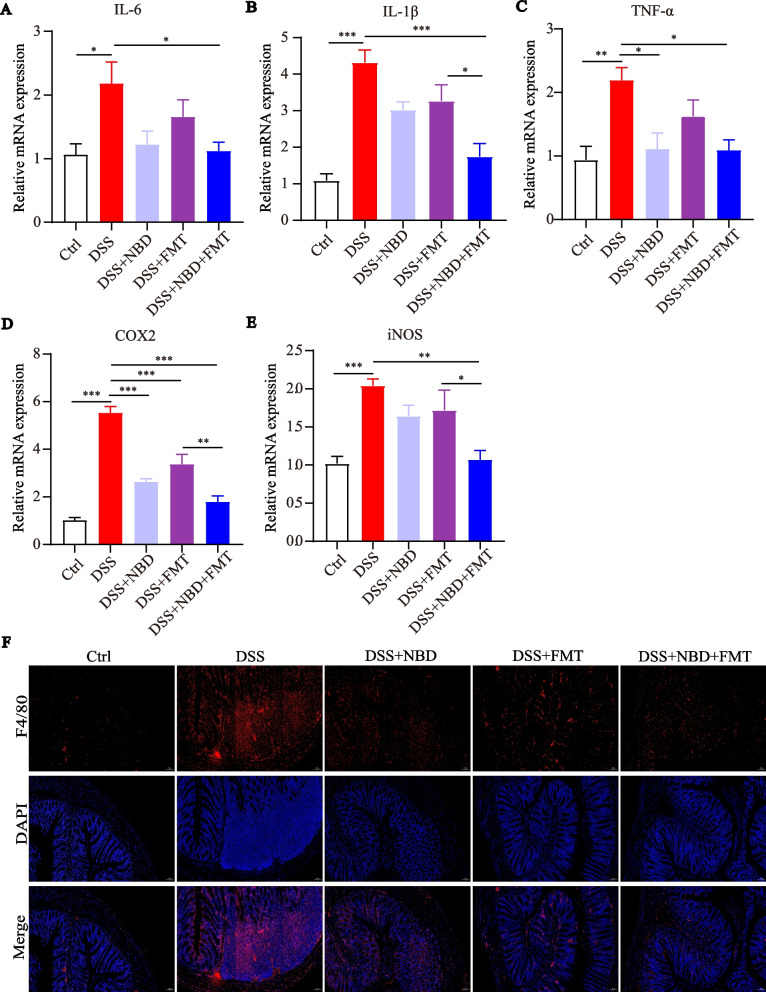
NBD combined with FMT inhibited inflammatory cytokines in rats with DSS-induced colitis. (**A-E**) NBD combined with FMT downregulates IL-6 (**A**), IL-1β (**B**), TNF-α (**C**), COX-2 (**D**) and iNOS (**E**) mRNA in colonic tissue of rats with UC. (**F**) Immunohistochemical staining showing F4/80 expression in rats’ colons. Data are expressed as mean ± SEM, *P < 0.05, **P < 0.01, ***P < 0.001

To understand the anti-inflammatory effects of NBD and FMT in rats with colitis, we measured the gene expression of pro-inflammatory cytokines and enzymes by PCR. Higher tumor necrosis factor (TNF)-α, interleukin (IL)-1β, IL-6, inducible nitric oxide synthetase (iNOS) and cyclooxygenase-2 (COX-2) levels were found in the colitic rats than rats in Ctrl group, but were dramatically downregulated by co-treatment with NBD and FMT (Fig. 4A-E). Compared with the DSS group, NBD significantly reduced the levels of TNF-α and COX-2 (*P* < 0.05 or *P* < 0.001), while FMT only downregulated the level of COX-2 (*P* < 0.001). In addition, the combination treatment was more effective in increasing IL-1β, iNOS and COX-2 levels compared with FMT treatment (*P* < 0.05 or *P* < 0.01). However, there was no significant difference in the effect of the combination therapy compared with NBD treatment (*P*>0.05). Macrophage infiltration is considered a parameter for evaluating the severity of UC [25]. NBD combined with FMT significantly decreased the percentage of F4/80^+^ cells (Fig. 4F). In a word, our data indicated that NBD combined with FMT in reducing inflammatory cytokines is more effective than either treatment alone.

### NBD combined with FMT suppressed the JAK2/STAT3/NF-κB signaling pathway in vivo

**Fig. 5:**
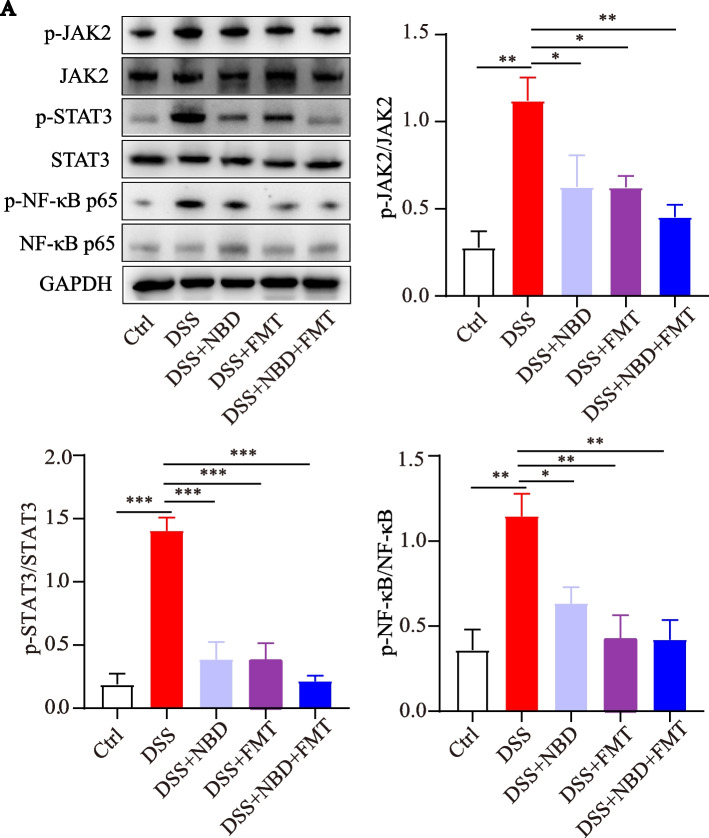
NBD combined with FMT suppressed the activity of the JAK2/STAT3/NF-κB pathway in rats with DSS-induced colitis. (**A**) Western blot images of p-JAK2, JAK2, p-STAT3, STAT3, p-NF-κB, NF-κB and GAPDH. The p-JAK2/JAK2, p-STAT3/STAT3 and p-NF-κB/NF-κB ratios are presented, *n* = 3. Data are expressed as mean ± SEM, *P < 0.05, **P < 0.01, ***P < 0.001

NF-κB regulates the expression of targeted inflammatory mediators, and JAK/STAT activity plays a crucial role in the pathogenesis of UC. We used protein blot analysis to investigate whether NBD combined with FMT might have therapeutic effects via the JAK2/STAT3/NF-κB signaling pathway. As shown in Fig. 5A, DSS-stimulated colitis rats showed a clear trend of upregulation of p-NF-κB p65, p-STAT3 and p-JAK2 proteins, all of which were downregulated in rats treated with three treatments (*P* < 0.05，*P* < 0.01 or *P* < 0.001). However, no significant differences were observed in the expression of JAK2, STAT3 and NF-κB p65 proteins between the NBD combined with FMT group and the other two groups (*P>*0.05).

### Composition analysis of the gut microbiota

**Fig. 6:**
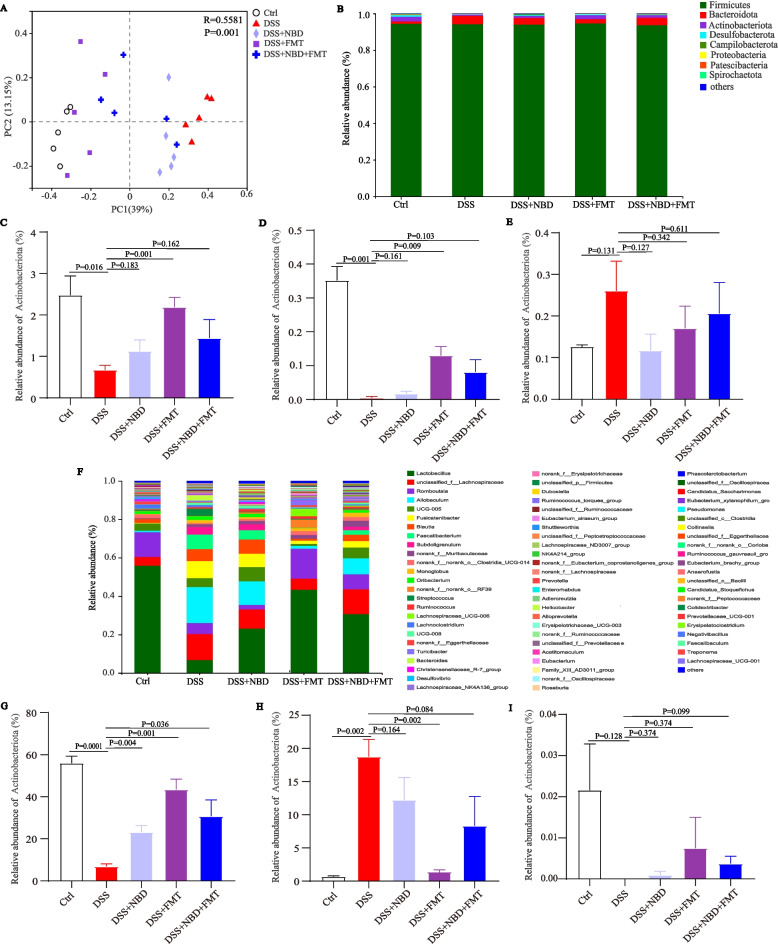
Composition analysis of the intestinal microbiota. (**A**) PCoA of gut microbiota communities on the basis of OTU levels, *n* = 5. (**B)** Bacterial compositions in each group of rats at the phylum level, *n* = 5. (**C-E**) Relative abundance of *Actinobacteria* (**C**), *Patescibacteria* (**D**) and *Proteobacteria* (**E**), *n* = 5. (**F**) Bacterial compositions in each group of rats at the genus level, *n* = 5. (**G-I**) Relative abundance of *Lactobacillus* (**G**)*, Allobaculum* (**H**) and *Akkermansia* (**I**), *n* = 5

The substantial separation of the Ctrl and DSS group on the two-dimensional coordinate map indicated some differences in the microbial structures between the groups, whereas the distance between samples indicated better reproducibility within the groups (Fig. 6A). The within-group reproducibility was poor in the NBD, FMT and combination group. However, the distance between the FMT group and the combination group was relatively close, and the two groups were distant from the DSS group. The bacterial composition of the FMT group was closest to that of the Ctrl group, followed by the combination treatment group, and that of the NBD group was relatively poor. The composition of the intestinal microflora in rats changed after treatment.

To further understand the effects of NBD combined with FMT on the gut microbiota, we investigated the composition of the gut microbiota at two taxonomic levels. The microbial community stacking histogram indicated that the fecal microorganisms in rats primarily comprised *Firmicutes*, *Bacteroidetes* and *Actinobacteria* at the phylum level (Fig. 6B). DSS intervention resulted in a clear decrease in the relative abundance of *Actinobacteria* and *Patescibacteria* (*P* < 0.001 or *P* < 0.05) (Fig. 6C,D). After FMT treatment, their relative abundance was significantly reversed. NBD combined with FMT upregulated the relative abundance of *Actinobacteria* and *Patescibacteria* but showed no statistical difference (*P>*0.05). Furthermore, NBD decreased the relative abundance of *Proteobacteria* below that in the DSS group (*P* = 0.127) (Fig. 6E). The genus-level intestinal flora was mainly composed of *Lactobacillus*, *unclassified_f__Lachnospiraceae*, *Romboutsia* and *Allobaculum* (Fig. 6F). Among these, the relative abundance of *Lactobacillus* was low in the DSS group (*P* < 0.001) (Fig. 6G). NBD, FMT and combination therapy alleviated the inhibition of the above bacterial genera (*P* < 0.001 or *P* < 0.05). In addition, the relative abundance of *Allobaculum* increased in the DSS group but decreased after treatment with FMT (*P* < 0.01) and NBD combined with FMT (*P* = 0.084) (Fig. 6H). Notably, the effect of FMT was better than that of combination treatment. Compared with that in rats with colitis, the relative abundance of *Akkermansia* was greater in the group with NBD combined with FMT (*P* = 0.099) (Fig. 6I).

### LEfse analysis of the intestinal flora in rats

**Fig. 7:**
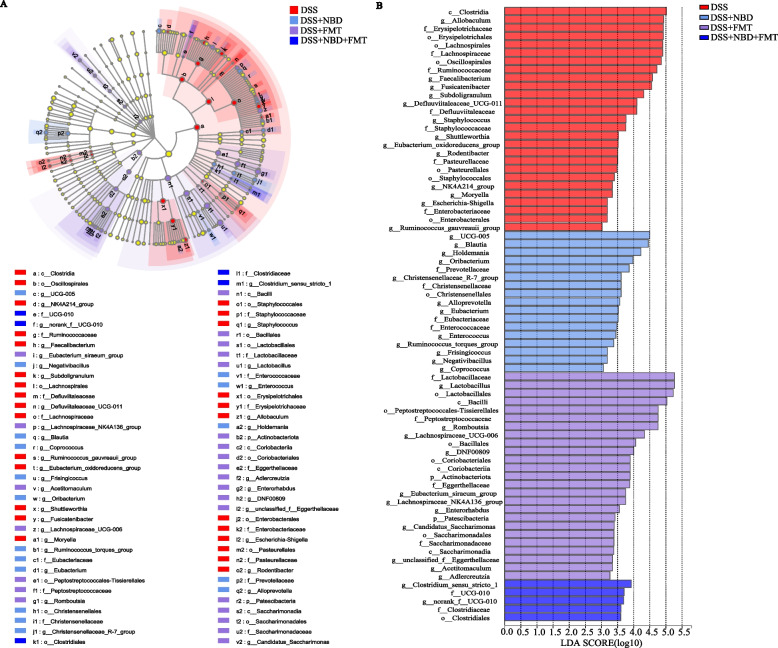
(**A,B**) Differences in dominant microorganisms among four groups, on the basis of a cladogram (**A**) and distribution histogram (**B**), with an LDA score larger than 2.0 as the criterion, *n* = 5

LEfSe is a statistical tool enabling the identification of taxa that may distinguish groups on the basis of biostatistical differences. Microorganisms with higher abundance were identified by LDA values greater than 2.0 as the criterion. As shown in our results, 27 taxa were identified in DSS-treated rats (Fig. 7A). A total of 17, 25 and 5 taxa were identified from rats with colitis treated with NBD, FMT, and NBD combined with FMT, respectively. According to the analysis results, *g__Allobaculumthe* and *g__Escherichia-Shigella* may be the key bacterial types responsible for the imbalance in the gut flora in the DSS group (Fig. 7B). The difference in the degree of contribution of *g_UCG-005*, *g_Blautia*, *g__Alloprevotell*, etc., was higher in the NBD group, and *g__Lactobacillus*, *g__Romboutsia*, *g__Lachnospiraceae_UCG-006*, *p__Actinobacteriota*, *g__Lachnospiraceae_NK4A136_group*, etc. were relatively prominent in the FMT group. Furthermore, *g__Clostridium_sensu_stricto_1*, *g__norank_f__UCG-010*, *f__Clostridiaceae*, etc. in the NBD combined with FMT group were significantly distinct from the microbiota in the other groups. In general, the diversity and composition of the gut microbiota were significantly transformed after the three different treatments.

### NBD combined with FMT increased SCFAs in rats with ulcerative colitis

**Fig. 8:**
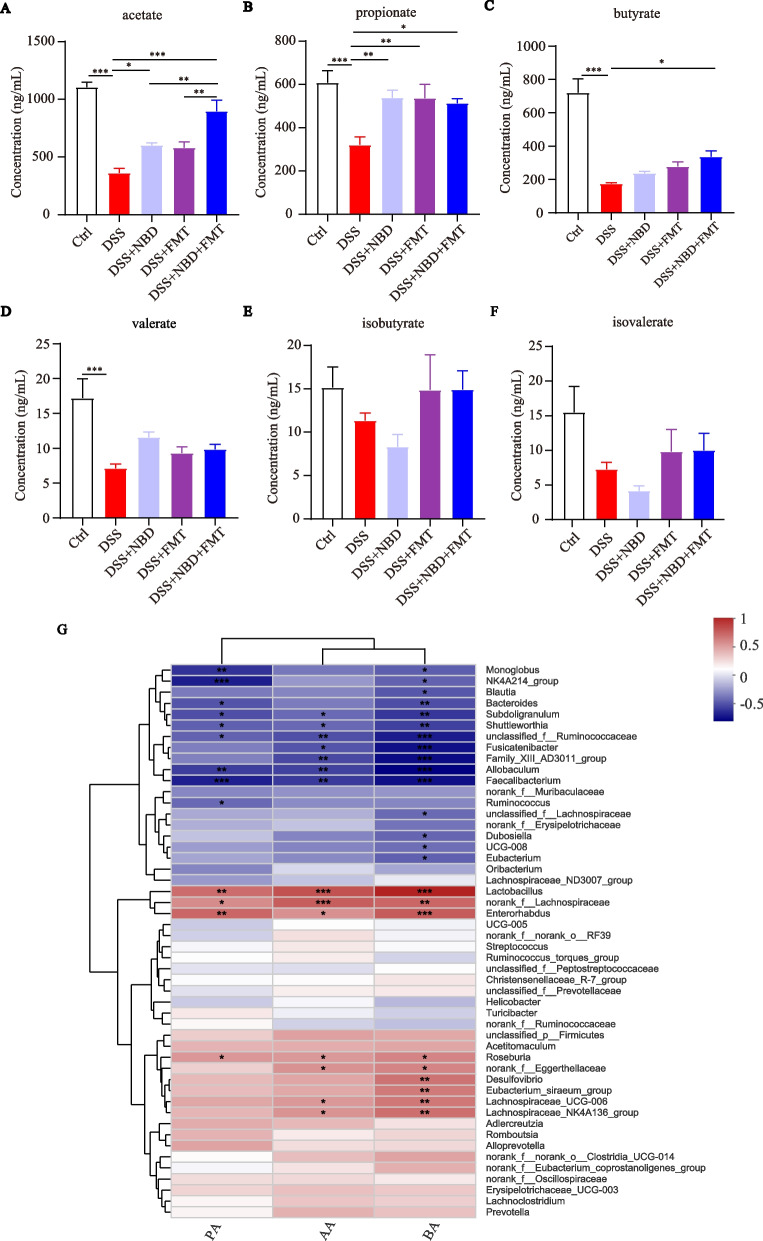
NBD combined with FMT increased SCFAs in the colonic feces of rats with colitis to strengthen the intestinal microbial balance. (**A-F**) The amounts of SCFAs, including acetate (**A**), propionate (**B**), butyrate (**C**), valerate (**D**), isobutyrate (**E**) and isovalerate (**F**), as detected by LC-MS, *n* = 6. Data are expressed as mean ± SEM, *P < 0.05, **P < 0.01, ***P < 0.001. (**G**) Heatmap showing Spearman correlations between several fecal SCFAs with significant differences and bacterial genera with a coefficient less than 0.05

SCFAs, metabolites of the gut microbiota, provide the primary energy source for colonocytes and play a role in intestinal homeostasis by inhibiting inflammation [26]. To confirm whether NBD in combination with FMT might affect SCFA metabolism, we determined the amounts of SCFAs in the colonic contents. The SCFA profiles of the DSS group were completely different from those of treatment groups: the content of acetate, butyrate, propionate and valerate acids were significantly lower in the rats with UC than the Ctrl group (*P* < 0.001) (Fig. 8A-D). NBD significantly upregulated the levels of propionate and acetate (*P* < 0.05 or *P* < 0.01), and FMT increased the level of propionate compared with the results for rats in the DSS group (*P* < 0.01). Also, combination treatment markedly upregulated the content of butyrate, propionate and acetate above that in rats with DSS-induced colitis (*P* < 0.001 or *P* < 0.05). Furthermore, the content of acetate was clearly higher in the combination group than the NBD and FMT groups (*P* < 0.01). The valerate, isovalerate and isobutyrate concentrations were slightly higher in the combination group than the DSS group, but were not statistically significantly different (*P*>0.05) (Fig. 8D-F).

Correlation heatmap analysis was used to determine the potential relationship between altered gut microflora and SCFAs. The result of the correlation heatmap analysis revealed a link between the gut microbiota and SCFAs (Fig. 8G). At the genus level, acetate, propionate and butyrate were positively correlated with *Lactobacillus*, *norank_f__Lachnospiraceae*, *Enterorhabdus* and *Roseburia*, but were negatively associated with *Subdoligranulum*, *Shuttleworthia*, *unclassified_f__Ruminococcaceae*, *Allobaculum* and *Faecalibacterium.*

## Discussion

To seek better treatments for UC, therapies such as traditional Chinese medicine, FMT and combination treatment are increasingly being used as adjuvant treatments. NBD, a clinical prescription used in clinical practice in China, is highly effective in light-to-moderate active UC. In addition, a variety of combination treatments have been demonstrated to be superior to monotherapy [27, 28]. Our results suggested that NBD in combination with FMT may have more significant therapeutic effects on colitis than either treatment alone. The body weight, DAI score and the shortening of the colon length were alleviated by NBD and FMT treatment. NBD and FMT mitigated the effects of DSS-induced colitis and may be used to prevent inflammatory bowel disease (IBD) in patients. The combination therapy performed better than the single treatments alone.

The intestinal microbiota plays a pivotal role in maintaining gastrointestinal health. Imbalances in the intestinal microflora can promote the development of UC, thus affecting the renewal of intestinal epithelial cells, intestinal peristalsis and metabolism, and mucosal and immune function [29, 30]. Therefore, investigating the roles of gut microflora in the pathogenesis of UC is crucial, to find effective ways to restore the intestinal microbial balance and alleviate UC. In our results, the diversity of the gut microbial community was significantly diminished in rats with DSS-induced colitis. The PCoA beta diversity revealed that the gut microbiota of colitic rats was clearly separate from that of rats in the Ctrl group, in agreement with a previous report [31]. The human gut microbiota is mainly assigned to four phyla. Rats have similar dominant bacteria to humans. The *Firmicutes* and *Bacteroidetes* phyla are the dominant microbiota, followed by the *Proteobacteria* and *Actinobacteria* phyla [32]. It was reported that the relative abundance of *Actinobacteria* significantly decreases with DSS [33]. *Actinobacteria*, which are gram positive anaerobic bacteria, maintain intestinal homeostasis [34]. In our results, the abundance of *Actinobacteria* remarkably reduced in the colitic rats, but increased in the FMT group. Meanwhile, FMT increased the relative abundance of the beneficial bacteria *Patescibacteria*. In our study, the level of *Escherichia-Shigella* was greater in the DSS group. *Shigella*, a genus in the *Enterobacteriaceae* family, is a crucial factor in increasing intestinal permeability and a major pathogen causing intestinal infection [35, 36]. Moreover, FMT treatment increased the abundance of *Lactobacillus* genera. *Lactobacillus*, characterized as a common probiotic, can enhance immunity, regulate the intestinal flora, and have anti-cancer and anti-diarrhea effects [37]. One study has shown that *Lactobacillus reuteri* ameliorates DSS-induced colitis in rats by increasing the colonic mucus thickness [38]. *Akkermansia* which belongs to the genus *Verruca*, interacts closely with hosts: it regulates mucus homeostasis of the host, and influences intestinal inflammation and immune system function [39]. All our gut microbiota results suggested that NBD, FMT and combination treatment maintained the gut microbial balance by interfering with different physiological processes through the gut flora. Notably, of the three treatments, FMT had the most significant role in the intestinal microbiota in UC, and was followed by NBD combined with FMT; NBD alone had the least clear role in influencing the intestinal microbiota.

SCFAs, important microbial metabolites, have anti-tumor and anti-inflammatory activity, and play essential roles in ameliorating enteritis, regulating the immune system, promoting intestinal epithelial barrier function and enhancing defense [40]. Therefore, the amounts of SCFAs were determined in this study. NBD and FMT increased the content of SCFAs in the intestinal tract in rats with UC to levels greater than those in the DSS group. Acetate, generated by probiotic *Lactobacillus*, can maintain the barrier function of the intestinal epithelium and protect the host from lethal infection [41]. Meanwhile, propionate and butyrate maintain intestinal health and delay the development of colitis [42]. Spearman correlation analysis showed that acetic acid, butyric acid and propionic acid were positively associated with *Lactobacillus*, *Roseburia* and *Enterorhabdus*. Our results revealed that *Lactobacillus*, *Roseburia* and *Enterorhabdus* were associated with the increase in SCFAs with NBD and FMT treatment, in agreement with findings from previous reports [43, 44]. We hypothesized that NBD influences the metabolism of gut microbiota, such as SCFA, which may be key to the treatment of UC, and that the exact mechanism of influence needs to be investigated in depth.

The development of colitis is known to increase the production of inflammatory cytokines and proinflammatory enzymes [9]. As expected, in our study, NBD combined with FMT decreased the mRNA expression of TNF-α, IL-1β, IL-6, iNOS and COX-2, thus indicating anti-inflammatory activity in UC. Also, the anti-inflammatory effect of the combination treatment was more pronounced than that of monotherapy. Macrophages, which are the major source of many inflammatory cytokines, spread in tissues throughout the body and are important effector cells of the innate immune system [45]. Tissue macrophages in IBD can significantly increase, thus leading to persistent inflammation. Immunofluorescence indicated that the presence of NBD in combination with FMT significantly inhibited the number of DSS-induced colon macrophages. NBD in combination with FMT inhibited macrophage activation, exerting anti-inflammatory activity.

NF-κB releases pro-inflammatory cytokines and induces proinflammatory enzymes [46]. We found that NBD combined with FMT also inhibited the phosphorylation of NF-κB p65 in the DSS-stimulated colon. Consequently, we hypothesized that the combination of NBD and FMT downregulated the expression of inflammatory cytokines and enzymes in DSS-stimulated colitis, a response potentially associated with inhibition of NF-κB activation. Furthermore, IL-6 activates STAT3, thus leading to elevated levels of anti-apoptotic factors induced by downstream STAT3 [47]. Inhibition of STAT3 ameliorates DSS-induced damage due to colon inflammation by downregulating pro-inflammatory cytokines. STAT3 is a key signaling molecule downstream of the JAK substrates and cytokine receptors, and JAK2/STAT3 signals are the major pathway for transcription factors involved in the pro-inflammatory cytokine response [48]. An important finding in our study was the decreased phosphorylation of JAK2/STAT3 after NBD combined with FMT treatment, thus suggesting that inhibition of the JAK/STAT pathway might be involved in the anti-colitis effect of combination therapy. These findings suggested that NBD in combination with FMT effectively inhibited JAK2/STAT3/NF-κB activation in the experimental UC model. Previous evidence has suggested that, the release of proinflammatory cytokines affects the expression of tight junction proteins [43]. Subsequently, the altered expression of tight junction proteins enhances intestinal permeability, thus leading to an inflammatory cascade [49].

Tight junction proteins are important for function gut barrier and permeability [50]. Accumulating evidence indicates that dysfunctioned intestinal mucosal barrier may lead to the progression of colitis. In our study, DSS-induced UC significantly decreased the colonic occludin and/or ZO-1 expression, but the levels of these proteins significantly increased after NBD and FMT co-treatment. The results suggested that treatment with NBD combined with FMT ameliorated colitis by promoting the repair of intestinal mucosa.

It is worth considering that, on the one hand, NBD treatment may affect the intestinal flora and subsequently the impact of FMT, and may also have a direct effect on inflammatory as well as immunomodulatory signaling pathways, producing more positive changes. On the other hand, FMT provided the opportunity for multiple effects involving bacterial antigens, interactions with the host and synthesis of SCFA when re-establishing the normal microbiome. Therefore, the mechanism of NBD combined with FMT treatment is complex, so the synergistic effects need further study.

## Conclusion

We found that NBD, FMT and combination treatment had therapeutic effects in rats with UC. The protective mechanism may be linked to regulation NF-κB/JAK2/STAT3 and restoration of the intestinal flora.

## Supplementary Information

**Additional file 1.**

## Data Availability

The raw data supporting the conclusions of this article will be made available by the authors upon request. The sequencing data used in this study are stored in NCBI SRA database (SUB11837168). https://www.ncbi.nlm.nih.gov/sra/PRJNA862306.

## Abbreviations

DSS: Dextran sulfate sodium
UC: Ulcerative colitis
FMT: Fecal microbiota transplantation
NBD: New Baitouweng Decoction
NF-κB: Nuclear factor-kappa B
IκB: Inhibitor of NF-κB
JAK: Janus kinase
STAT: Signal transduction and transcriptional activator
DAI: Disease activity index
H&E: Hematoxylin and eosin
PBS: Phosphate buffered saline
qRT-PCR: Quantitative real-time polymerase chain reaction
GAPDH: Glyceraldehyde-3-phosphate dehydrogenase
IL-1β: Interleukin-1β
IL-6: Interleukin-6
TNF-α: Tumor necrosis factor-α
iNOS: Inducible nitric oxide synthetase
COX-2: Cyclooxygenase-2
ZO-1: Zona occludens 1
PVDF: Polyvinylidene fluoride
p-STAT3: phospho-STAT3
p-NF-κB: Phospho-NF-κB
p-JAK: Phospho-JAK
DAPI: 4′6-diamidino-2-phenylindole
SCFA: Short-chain fatty acid
LC-MS: Liquid chromatograph-mass spectrometer
OTU: Observed taxonomic unit
PCoA: Principal coordinate analysis
LDA: Line discriminant analysis
LEfSe: Line discriminant analysis effect size
IBD: Inflammatory bowel disease

## References

[1] Kucharzik T, Koletzko S, Kannengiesser K, Dignass A (2020). Ulcerative colitis-diagnostic and therapeutic algorithms. Dtsch Arztebl Int.

[2] Feuerstein JD, Moss AC, Farraye FA (2019). Ulcerative colitis. Mayo Clin Proc.

[3] Hoekman DR, Stibbe JA, Baert FJ, Caenepeel P, Vergauwe P, De Vos M (2018). Long-term outcome of early combined immunosuppression versus conventional Management in Newly Diagnosed Crohn's disease. J Crohns Colitis.

[4] Ishikawa D, Sasaki T, Osada T, Kuwahara-Arai K, Haga K, Shibuya T (2017). Changes in intestinal microbiota following combination therapy with fecal microbial transplantation and antibiotics for ulcerative colitis. Inflamm Bowel Dis.

[5] Gao W, Zhang L, Wang X, Yu L, Wang C, Gong Y (2018). The combination of indirubin and isatin attenuates dextran sodium sulfate induced ulcerative colitis in mice. Biochem Cell Biol.

[6] Guo XY, Liu XJ, Hao JY (2020). Gut microbiota in ulcerative colitis: insights on pathogenesis and treatment. J Dig Dis.

[7] Ji J, Ge X, Chen Y, Zhu B, Wu Q, Zhang J (2019). Daphnetin ameliorates experimental colitis by modulating microbiota composition and Treg/Th17 balance. FASEB J.

[8] Tian C, Huang Y, Wu X, Xu C, Bu H, Wang H (2020). The efficacy and safety of Mesalamine and probiotics in mild-to-moderate ulcerative colitis: a systematic review and Meta-analysis. Evid Based Complement Alternat Med.

[9] Peng L, Gao X, Nie L, Xie J, Dai T, Shi C (2020). Astragalin attenuates dextran sulfate sodium (DSS)-induced acute experimental colitis by alleviating gut microbiota Dysbiosis and inhibiting NF-kappaB activation in mice. Front Immunol.

[10] Banerjee S, Biehl A, Gadina M, Hasni S, Schwartz DM (2017). JAK-STAT signaling as a target for inflammatory and autoimmune diseases: current and future prospects. Drugs..

[11] Huangfu S, Dou R, Zhong S, Guo M, Gu C, Jurczyszyn A (2020). Modified Pulsatillae decoction inhibits DSS-induced ulcerative colitis in vitro and in vivo via IL-6/STAT3 pathway. BMC Complement Med Ther.

[12] Xuan-Qing C, Xiang-Yu LV, Shi-Jia LIU (2021). Baitouweng decoction alleviates dextran sulfate sodium-induced ulcerative colitis by regulating intestinal microbiota and the IL-6/STAT3 signaling pathway. J Ethnopharmacol.

[13] Guazelli CFS, Fattori V, Ferraz CR, Borghi SM, Casagrande R, Baracat MM (2021). Antioxidant and anti-inflammatory effects of hesperidin methyl chalcone in experimental ulcerative colitis. Chem Biol Interact.

[14] Gao X, Tang Y, Lei N, Luo Y, Chen P, Liang C (2021). Symptoms of anxiety/depression is associated with more aggressive inflammatory bowel disease. Sci Rep.

[15] Miao Z, Wang X, Zhang X, Zhang Y, Xu Y (2021). Clinical efficacy assessment of new Baitouweng decoction in the treatment of active ulcerative colitis. Zhongguo Zhong Xi Yi Jie He Za Zhi.

[16] Jia J, Zheng K, Shen H, Yu J, Zhu P, Yan S (2020). Qingchang Huashi granule ameliorates experimental colitis via restoring the dendritic cell-mediated Th17/Treg balance. BMC Complement Med Ther.

[17] Shen H, Liu ZQ, Zhu Q, Zhu L, Zhai JH (2013). Effect of qingchang huash recipe on NF-kappaB/Tolls pathway in ulcerative colitis patients and mechanism study. Zhongguo Zhong Xi Yi Jie He Za Zhi Zhongguo Zhongxiyi Jiehe Zazhi.

[18] Hua YL, Ma Q, Li W, Zhang XS, Cheng XH, Jia YQ (2019). Metabolomics analysis of Pulsatilla decoction on treatment of wetness-heat-induced diarrhea in rats based on UPLC-Q/TOF-MS/MS. Biomed Chromatogr.

[19] Chen Y, Miao Z, Sheng X, Li X, Ma J, Xu X (2022). Sesquiterpene lactones-rich fraction from Aucklandia lappa Decne. Alleviates dextran sulfate sodium induced ulcerative colitis through co-regulating MAPK and Nrf2/Hmox-1 signaling pathway. J Ethnopharmacol.

[20] Gu P, Zhu L, Liu Y, Zhang L, Liu J, Shen H (2017). Protective effects of paeoniflorin on TNBS-induced ulcerative colitis through inhibiting NF-kappaB pathway and apoptosis in mice. Int Immunopharmacol.

[21] Muluye RA, Bian Y, Alemu PN (2014). Anti-inflammatory and antimicrobial effects of heat-clearing Chinese herbs: a current review. J Tradit Complement Med.

[22] Hu J, Huang H, Che Y, Ding C, Zhang L, Wang Y (2021). Qingchang Huashi formula attenuates DSS-induced colitis in mice by restoring gut microbiota-metabolism homeostasis and goblet cell function. J Ethnopharmacol.

[23] Chiu CJ, McArdle AH, Brown R, Scott HJ, Gurd FN (1970). Intestinal mucosal lesion in low-flow states. I. a morphological, hemodynamic, and metabolic reappraisal. Arch Surg (Chicago, Ill : 1960).

[24] Han J, Lin K, Sequeira C, Borchers CH (2015). An isotope-labeled chemical derivatization method for the quantitation of short-chain fatty acids in human feces by liquid chromatography-tandem mass spectrometry. Anal Chim Acta.

[25] Lu N, Wang L, Cao H, Liu L, Van Kaer L, Washington MK (2014). Activation of the epidermal growth factor receptor in macrophages regulates cytokine production and experimental colitis. J Immunol.

[26] Correa-Oliveira R, Fachi JL, Vieira A, Sato FT, Vinolo MA (2016). Regulation of immune cell function by short-chain fatty acids. Clin Transl Immunol.

[27] Targownik LE, Benchimol EI, Bernstein CN, Singh H, Tennakoon A, Zubieta AA (2020). Combined biologic and Immunomodulatory therapy is superior to Monotherapy for decreasing the risk of inflammatory bowel disease-related complications. J Crohns Colitis.

[28] Tang S, Liu W, Zhao Q, Li K, Zhu J, Yao W (2021). Combination of polysaccharides from Astragalus membranaceus and Codonopsis pilosula ameliorated mice colitis and underlying mechanisms. J Ethnopharmacol.

[29] Zhang XJ, Yuan ZW, Qu C, Yu XT, Huang T, Chen PV (2018). Palmatine ameliorated murine colitis by suppressing tryptophan metabolism and regulating gut microbiota. Pharmacol Res.

[30] Lin R, Piao M, Song Y (2019). Dietary Quercetin increases colonic microbial diversity and attenuates colitis severity in Citrobacter rodentium-infected mice. Front Microbiol.

[31] Cui H, Cai Y, Wang L, Jia B, Li J, Zhao S (2018). Berberine regulates Treg/Th17 balance to treat ulcerative colitis through modulating the gut microbiota in the Colon. Front Pharmacol.

[32] Ni J, Wu GD, Albenberg L, Tomov VT (2017). Gut microbiota and IBD: causation or correlation?. Nat Rev Gastroenterol Hepatol.

[33] Yang H, Cai R, Kong Z, Chen Y, Cheng C, Qi S (2020). Teasaponin ameliorates murine colitis by regulating gut microbiota and suppressing the immune system response. Front Med (Lausanne).

[34] Binda C, Lopetuso LR, Rizzatti G, Gibiino G, Cennamo V, Gasbarrini A (2018). Actinobacteria: a relevant minority for the maintenance of gut homeostasis. Dig Liver Dis.

[35] Bian X, Wu W, Yang L, Lv L, Wang Q, Li Y (2019). Administration of Akkermansia muciniphila ameliorates dextran sulfate sodium-induced ulcerative colitis in mice. Front Microbiol.

[36] Nisa I, Qasim M, Yasin N, Ullah R, Ali A (2020). Shigella flexneri: an emerging pathogen. Folia Microbiol (Praha).

[37] de Vos P, Mujagic Z, de Haan BJ, Siezen RJ, Bron PA, Meijerink M (2017). Lactobacillus plantarum strains can enhance human mucosal and systemic immunity and prevent non-steroidal anti-inflammatory drug induced reduction in T regulatory cells. Front Immunol.

[38] Ahl D, Liu H, Schreiber O, Roos S, Phillipson M, Holm L (2016). Lactobacillus reuteri increases mucus thickness and ameliorates dextran sulphate sodium-induced colitis in mice. Acta Physiol (Oxford).

[39] Zhang T, Li Q, Cheng L, Buch H, Zhang F (2019). Akkermansia muciniphila is a promising probiotic. Microb Biotechnol.

[40] Li M, van Esch B, Wagenaar GTM, Garssen J, Folkerts G, Henricks PAJ (2018). Pro- and anti-inflammatory effects of short chain fatty acids on immune and endothelial cells. Eur J Pharmacol.

[41] Adamberg S, Sumeri I, Uusna R, Ambalam P, Kondepudi KK, Adamberg K, et al. Survival and synergistic growth of mixed cultures of bifidobacteria and lactobacilli combined with prebiotic oligosaccharides in a gastrointestinal tract simulator. Microb Ecol Health Dis. 2014:25. 10.3402/mehd.v25.23062 PMC4101457 25045346

[42] Cui L, Guan X, Ding W, Luo Y, Wang W, Bu W (2021). Scutellaria baicalensis Georgi polysaccharide ameliorates DSS-induced ulcerative colitis by improving intestinal barrier function and modulating gut microbiota. Int J Biol Macromol.

[43] Wang MX, Lin L, Chen YD, Zhong YP, Lin YX, Li P (2020). Evodiamine has therapeutic efficacy in ulcerative colitis by increasing Lactobacillus acidophilus levels and acetate production. Pharmacol Res.

[44] Li Q, Cui Y, Xu B, Wang Y, Lv F, Li Z (2021). Main active components of Jiawei Gegen Qinlian decoction protects against ulcerative colitis under different dietary environments in a gut microbiota-dependent manner. Pharmacol Res.

[45] Oishi Y, Manabe I (2018). Macrophages in inflammation, repair and regeneration. Int Immunol.

[46] Yang L, Liu G, Lian K, Qiao Y, Zhang B, Zhu X (2019). Dietary leonurine hydrochloride supplementation attenuates lipopolysaccharide challenge-induced intestinal inflammation and barrier dysfunction by inhibiting the NF-kappaB/MAPK signaling pathway in broilers. J Anim Sci.

[47] Chu XQ, Wang J, Chen GX, Zhang GQ, Zhang DY, Cai YY (2018). Overexpression of microRNA-495 improves the intestinal mucosal barrier function by targeting STAT3 via inhibition of the JAK/STAT3 signaling pathway in a mouse model of ulcerative colitis. Pathol Res Pract.

[48] Xin P, Xu X, Deng C, Liu S, Wang Y, Zhou X, et al. The role of JAK/STAT signaling pathway and its inhibitors in diseases. Int Immunopharmacol. 2020;80. 10.1016/j.intimp.2020.106210 31972425

[49] Ogata M, Ogita T, Tari H, Arakawa T, Suzuki T (2017). Supplemental psyllium fibre regulates the intestinal barrier and inflammation in normal and colitic mice. Br J Nutr.

[50] Zeisel MB, Dhawan P, Baumert TF (2019). Tight junction proteins in gastrointestinal and liver disease. Gut..

