# Supplementary material for: New Baitouweng decoction combined with fecal microbiota transplantation alleviates DSS-induced colitis in rats by regulating gut microbiota metabolic homeostasis and the STAT3/NF-κB signaling pathway
Source: BMC Complement Med Ther. 2022 Nov 24;22:307. doi: 10.1186/s12906-022-03766-z (PMC9686021; doi:10.1186/s12906-022-03766-z)

****Supplementary Table S1**. Criteria of Chiu’s grading in the evaluation of intestinal mucosal injury.**

| Fraction Grade Standard |
| --- |
| 1 Grade 0 Normal intestinal villi  2 Grade 1 Submucosal spaces on the top of villi,capillary filled with blood  3 Grade 2 Separation of the intestinal mucosa from the submucosa  4 Grade 3 Separation of the intestinal mucosa from the submucosa extending to the sides of the intestinal villi  5 Grade 4 The villi becomig blunted, Exposure of laminae propria and blood vessels, Inflammatory tissue infiltration  6 Grade 5 Lamina propria digestion and decomposition,bleeding or forming a ulceration |

****Supplementary Table S2****. **Primers used in the real-time PCR assays.**

| TNF-α  Forward  Reverse  IL-1β  Forward  Reverse  IL-6  Forward  Reverse  iNOS  Forward  Reverse  COX2  Forward  Reverse  Occludin  Forward  Reverse  ZO-1  Forward  Reverse  GAPDH  Forward  Reverse | GGCATGGATCTCAAAGACAACC  AAATCGGCTGACGGTGTGG  TCTCACAGCAGCATCTCGAC  GGTCGTCATCATCCCACGAG  CTGCTCTGGTCTTCTGGAGT  GCATTGGAAGTTGGGGTAGG  GACCAAACTGTGTGCCTGGA  TACTCTGAGGGCTGACACAAGG  GCATTCTTTGCCCAGCACTT  GTCTTTGACTGTGGGAGGAT  CCAATCATTATGCACCAAGC  GAATTTCGTCTTCCGGGTAA  AAGACAGCCTCTCAACAGAAAGC  AGCTTGTGATACGTGCGAGG  CAAGTTCAACGGCACAGTCAAG  ACATACTCAGCACCAGCATCAC |
| --- | --- |

****Supplementary Table S3****. The identified main chemical components of NBD with analyse retention times, precise molecular ion peaks and secondary mass spectrometry cleavage fragments.

| NO. | RT(min) | Identification | Formula | Mass  (Da) | Adduct | Extraction Mass | Found At Mass (Da) | Error (ppm) | Intensity | MS/MS fragments |
| --- | --- | --- | --- | --- | --- | --- | --- | --- | --- | --- |
| 1 | 0.56 | Glucogallin | C13H16O10 | 332.07435 | [M-H]- | 331.0675 | 331.06737 | -0.4 | 178814 | 331.0662,271.0451,169.0139 |
| 2 | 0.9 | Gallic acid glycoside | C13H16O10 | 332.07435 | [M-H]- | 331.06707 | 331.06737 | 0.9 | 178814 | 271.0451, 211.0247,169.0139，125.0243 |
| 3 | 0.95 | Oryzanolone - 1- O- β- D- glucoside | C16H24O9 | 360.14203 | [M+H]+ | 361.14931 | 361.14947 | 0.4 | 25805 | 361.1360，163.0763,85.0336 |
| 4 | 1.02 | Gallic acid | C7H6O5 | 170.02152 | [M-H]- | 169.01425 | 169.01511 | 5.1 | 1395439 | 169.0126,125.0234 |
| 5 | 1.4 | Benzoic acid, 4-(b-D-glucopyranosyloxy)-3,5-dimethoxy- | C15H20O10 | 360.10565 | [M-H]- | 359.09837 | 359.09824 | -0.4 | 266559 | 359.0982，197.0451 |
| 6 | 2.05 | Protocatechuic acid | C7H6O4 | 154.02661 | [M-H]- | 153.01933 | 153.02026 | 6.1 | 309781 | 153.0184,109.0285 |
| 7 | 3.37 | Paeonol | C9H10O3 | 166.06299 | [M-H]- | 165.05572 | 165.05647 | 4.5 | 155044 | 165.0531,122.0645 |
| 8 | 3.77 | Esculin | C15H16O9 | 340.07943 | [M-H]- | 339.07216 | 339.07229 | 0.4 | 280701 | 177.0184,133.0290 |
| 9 | 4.87 | Vanillic acid | C8H8O4 | 168.04226 | [M-H]- | 167.03498 | 167.03574 | 4.5 | 49340 | 167.0357,152.0099,123.0447 |
| 10 | 5.24 | Epicatechin | C15H14O6 | 290.07904 | [M-H]- | 289.07176 | 289.07196 | 0.7 | 268618 | 271.0598, 245.0805, 203.0700,161.0598 |
| 11 | 5.55 | aesculetin | C9H6O4 | 178.02661 | [M-H]- | 177.01933 | 177.02017 | 4.7 | 590745 | 149.0238，133.0289,107.0133 |
| 12 | 5.76 | Caffeic acid | C9H8O4 | 180.04226 | [M-H]- | 179.03498 | 179.03534 | 2.0 | 204177 | 179.0344,135.0448,89.0409 |
| 13 | 5.77 | Paeoniflorin oxide | C23H28O12 | 496.15808 | [M-H]- | 495.1508 | 495.15 | -1.6 | 809785 | 495.1496，345.1186 |
| 14 | 6.02 | Chlorogenic acid | C16H18O9 | 354.09508 | [M-H]- | 353.08781 | 353.08757 | -0.7 | 404824 | 353.0865,191.0551,173.0445,135.0442 |
| 15 | 6.14 | Brevifolincarboxylic acid | C13H8O8 | 292.02192 | [M-H]- | 291.01464 | 291.01496 | 1.1 | 365491 | 247.0232, 203.0343,147.0439 |
| 16 | 6.61 | Hexylamine tétrahydroafricaine | C20H23NO4 | 341.16271 | [M+H]+ | 342.16998 | 342.1701 | 0.4 | 2438405 | 342.1674,192.0998,177.0763 |
| 17 | 6.62 | Fraxin | C16H18O10 | 370.09 | [M-H]- | 369.08272 | 369.08268 | -0.1 | 519300 | 207.0287,192.0055,163.0032,135.0089 |
| 18 | 6.87 | Trigonelloside | C27H24O18 | 636.09626 | [M-H]- | 635.08899 | 635.08852 | -0.7 | 186686 | 635.0904,483.0778, 465.0688,423.0566,313.0578，295.0447 |
| 19 | 7.29 | Fraxetin | C10H8O5 | 208.03717 | [M-H]- | 207.0299 | 207.03033 | 2.1 | 64853 | 192.0062,175.0029 |
| 20 | 7.43 | 7-Methoxycoumarin | C10H8O3 | 176.04734 | [M+H]+ | 177.05462 | 177.05437 | -1.4 | 1190746 | 145.0283,117.0343 |
| 21 | 7.46 | Paeonolide | C20H28O12 | 460.15808 | [M-H]- | 459.1508 | 459.15041 | -0.8 | 11837 | 459.1424,293.0867 |
| 22 | 7.47 | Paeonolactone glucoside | C23H28O11 | 480.16316 | [M-H]- | 479.15589 | 479.1544 | -3.1 | 471892 | 479.1564,121.0293 |
| 23 | 7.66 | Corydaldine | C11H13NO3 | 207.08954 | [M-H]- | 206.08227 | 206.0823 | 0.1 | 12540 | 206.0821,164.0710, 147.0444, 103.0546, 91.0572 |
| 24 | 7.79 | 5-O-Feruloylquinic acid | C17H20O9 | 368.11073 | [M-H]- | 367.10346 | 367.10315 | -0.8 | 4363073 | 193.0483,191.0542,173.0435 |
| 25 | 8.14 | Anemonin | C10H8O4 | 192.04226 | [M+H]+ | 193.04954 | 193.04929 | -1.3 | 193.04929 | 193.0486,178.0251,165.0537,137.0595 |
| 26 | 8.33 | Noroxyhydrastinine | C10H9NO3 | 191.05824 | [M+H]+ | 192.06552 | 192.0658 | 1.5 | 26934 | 192.0644, 174.0540, 163.0370 |
| 27 | 8.33 | Dihydrokaempferol | C15H12O6 | 288.06339 | [M+H]+ | 289.07066 | 289.07081 | 0.5 | 207833 | 271.0590,215.0701,153.0182 |
| 28 | 8.57 | Ferulic acid | C10H10O4 | 194.05791 | [M-H]- | 193.05063 | 193.05121 | 3.0 | 255022 | 178.0263,149.0598,134.0367 |
| 29 | 8.64 | Tetrahydropalmatine | C21H25NO4 | 355.17836 | [M+H]+ | 356.18564 | 356.1855 | -0.4 | 291225 | 356.1864,190.0783 |
| 30 | 8.64 | Perillic acid | C27H22O12 | 538.11113 | [M-H]- | 537.10385 | 537.10382 | -0.1 | 15660 | 537.1213,295.0600 |
| 31 | 9.04 | Protopine | C20H19NO5 | 353.12632 | [M+H]+ | 354.1336 | 354.1339 | 0.8 | 7467 | 354.1353, 339.1036, 324.0807, 310.1083 |
| 32 | 9.07 | Liquiritin | C21H22O9 | 418.12638 | [M-H]- | 417.11911 | 417.1181 | -2.4 | 898646 | 417.1167,255.0652,135.0085 |
| 33 | 9.14 | Rhynchophylline | C14H6O8 | 302.00627 | [M-H]- | 300.99899 | 300.9993 | 1.0 | 478102 | 283.9942, 257.0073,229.0122,201.0174,185.0226 |
| 34 | 9.15 | Glycyrrhizin-4'-apigenin | C26H30O13 | 550.16864 | [M-H]- | 549.16137 | 549.1602 | -2.1 | 1036546 | 549.1600,255.0651,135.0081 |
| 35 | 9.53 | Gallic acid paeoniflorin | C30H32O15 | 632.17412 | [M-H]- | 631.16684 | 631.16626 | -0.9 | 1562034 | 613.1542,509.1285,313.0548,169.0131 |
| 36 | 9.73 | Dilactone digluconate | C21H10O13 | 470.01214 | [M-H]- | 469.00486 | 469.00445 | -0.9 | 34798 | 314.9768, 300.9986,286.9801 |
| 37 | 9.75 | Oxyberberine | C20H17NO5 | 351.11067 | [M+H]+ | 352.11795 | 352.1176 | -1.0 | 179801 | 352.1176, 337.0940, 322.0703, 308.0914, 294.0716 |
| 38 | 10.17 | Mudanpioside H | C30H32O14 | 616.17921 | [M-H]- | 615.17193 | 615.17138 | -0.9 | 127264 | 585.1591,431.1342,281.0660,137.0234 |
| 39 | 10.56 | Dihydroberberine | C20H19NO4 | 337.13141 | [M+H]+ | 338.13868 | 338.1395 | 2.4 | 815148 | 322. 1063,307.0838,279.0882 |
| 40 | 10.73 | [jateorhizine](../Program Files (x86)/Youdao/Dict/8.10.3.0/resultui/html/index.html" \l "\\javascript:;" \o "file:///D:\\Program Files (x86)\\Youdao\\Dict\\8.10.3.0\\resultui\\html\\index.html#\\javascript:;) | C20H19NO4 | 337.13141 | [M+H]+ | 338.13868 | 338.13888 | 0.6 | 815148 | 322. 1063,307.0822,279.0873 |
| 41 | 10.95 | Canadine | C20H21NO4 | 339.14706 | [M-H]- | 338.13978 | 338.1407 | 2.7 | 3486 | 338.1384, 323.1129, 308.0935，264.0676 |
| 42 | 11.07 | Alkannin | C16H16O5 | 288.09977 | [M-H]- | 287.0925 | 287.09236 | -0.5 | 19010 | 287.0544,243.1029,149.0244 |
| 43 | 11.15 | 2-(4-Hydroxyphenyl)-7-[(2S,3R,4S,5S,6R)-3,4,5-trihydroxy-6-(hydroxymethyl)oxan-2-yl]oxy-2,3-dihydrochromen-4-one | C21H22O9 | 418.12638 | [M+H]+ | 419.13366 | 419.13359 | -0.2 | 146811 | 257.0807,147.0437 |
| 44 | 11.41 | Isoliquiritin | C21H22O9 | 418.12638 | [M-H]- | 417.11911 | 417.119 | -0.3 | 898646 | 417.1191,255.0662,135.0081 |
| 45 | 11.76 | Paeonia New Sweet | C23H26O10 | 462.1526 | [M-H]- | 461.14532 | 461.14481 | -1.1 | 27860 | 329.0308,299.0926,193.0495 |
| 46 | 11.78 | wogonin | C16H14O5 | 286.08412 | [M-H]- | 285.07685 | 285.0767 | -0.5 | 33922 | 270.0525,177.0185，150.0313 |
| 47 | 11.83 | Liquiritigenin | C15H12O4 | 256.07356 | [M-H]- | 255.06628 | 255.0666 | 1.3 | 443037 | 255.0649,135.0080,119.0946 |
| 48 | 11.94 | [berberine](../Program Files (x86)/Youdao/Dict/8.10.3.0/resultui/html/index.html" \l "\\javascript:;" \o "file:///D:\\Program Files (x86)\\Youdao\\Dict\\8.10.3.0\\resultui\\html\\index.html#\\javascript:;) | C20H17NO4 | 335.11576 | [M+H]+ | 336.12303 | 336.12299 | -0.1 | 6328940 | 320.0887,292.0938，278.0782 |
| 49 | 11.95 | Fenhexidine | C21H21NO4 | 351.14706 | [M+H]+ | 352.15433 | 352.15442 | 0.3 | 1133288 | 336.1216,308.1266,294.1110 |
| 50 | 12.34 | quercetin | C15H10O7 | 302.04265 | [M-H]- | 301.03538 | 301.0354 | 0.1 | 126019 | 301.0338,151.0033,121.0291,107.0142 |
| 51 | 13.08 | Leiyemudanoside B | C59H96O27 | 1236.6139 | [M-H]- | 1235.60662 | 1235.609 | 1.9 | 30299 | 1235.6093,765.4441,469.1572 |
| 52 | 13.55 | naringenin | C15H12O5 | 272.06847 | [M-H]- | 271.0612 | 271.0619 | 2.6 | 87507 | 271.0604,151.0032，119.0498 |
| 53 | 13.84 | Anemoside B4 | C59H96O26 | 1220.61898 | [M-H]- | 1219.61171 | 1219.6139 | 1.8 | 10639 | 1219.6174,749.4492,469.1539 |
| 54 | 13.86 | Costunolide | C15H20O2 | 232.14633 | [M+H]+ | 233.15361 | 233.15341 | -0.9 | 512549 | 233.1523,215.1426,197.1323,187.1474 |
| 55 | 13.9 | Gamma-Piperine | C13H11NO3 | 229.07389 | [M+H]+ | 230.08117 | 230.08102 | -0.7 | 36446 | 172.0293,158.0585,116.0500 |
| 56 | 13.90 | hederagenin 3-O-{β-D-glucopyranosyl(1→2)[β-D-glucopyranosyl(1→4)]-α-L-arabinopyranoside} | C47H76O18 | 928.50317 | [M-H]- | 927.49589 | 927.4912 | -5.1 | 3337 | 927.3024,603.3880,471.3450 |
| 57 | 14.05 | Arbusculin A | C15H22O3 | 250.15689 | [M+H]+ | 251.16417 | 251.16423 | 0.2 | 26788 | 251.1639,233.1545 |
| 58 | 14.05 | 3-O-α-L-arabinopyranosyl-23-hydroxybetulinic acid 28-Oα-L-rhamnopyranosyl(1→4)-β-D-glucopyranosyl(1  →6)-β-D-glucopyranosyl ester | C53H86O22 | 1074.56108 | [M-H]- | 1073.5538 | 1073.5540 | 0.2 | 31247 | 1073.5589,603.3909,469.1562 |
| 59 | 14.56 | Pulsatilloside C | C48H78O18 | 942.51882 | [M-H]- | 941.51154 | 941.51068 | -0.9 | 22533 | 941.5107,733.4546,455.3482 |
| 60 | 14.58 | hederasaponin B | C59H96O25 | 1204.62407 | [M-H]- | 1203.61679 | 1203.6152 | -1.3 | 12822 | 1203.6236,733.4654,469.1549 |
| 61 | 14.75 | *Z-butenylphthalide* | C12H12O2 | 188.08373 | [M+H]+ | 189.09101 | 189.09077 | -1.3 | 52783 | 171.0824,143.0849,128.0629,115.0548 |
| 62 | 15.04 | Anemoside B7 | C47H76O16 | 896.51334 | [M-H]- | 895.50606 | 895.5087 | 2.9 | 35175 | 895.5098,733.4547,587.3951,455.3498 |
| 63 | 15.09 | Farrerol | C17H16O5 | 300.09977 | [M-H]- | 299.0925 | 299.09249 | 0.0 | 38160 | 217. 0449，187. 0450 |
| 64 | 15.11 | Deoxynivalenolide | C30H46O3 | 454.3447 | [M+H]+ | 455.35197 | 455.35124 | -1.6 | 1277046 | 455.3504,201.1628，119.0863 |
| 65 | 15.15 | Isoliquiritigenin | C15H12O4 | 256.07356 | [M-H]- | 255.06628 | 255.0671 | 3.2 | 443037 | 255.0644,135.0083,119.0501 |
| 66 | 15.31 | Diosgenin I | C35H56O8 | 604.39752 | [M-H]- | 603.39024 | 603.38926 | -1.6 | 162861 | 603.3901, 585.3788 |
| 67 | 15.33 | Pulsatilla B | C41H66O13 | 766.45034 | [M-H]- | 765.44307 | 765.44232 | -1.0 | 21668 | 765.4413,603.4185,471.3615 |
| 68 | 15.48 | 18-βglycyrrhetinic acid | C30H46O4 | 470.33961 | [M-H]- | 469.33233 | 469.33047 | -4.0 | 571882 | 469.3305217.1583 |
| 69 | 15.76 | Dehydrocostus lactone | C15H20O2 | 232.14633 | [M+H]+ | 233.15361 | 233.1533 | -1.3 | 512549 | 233.1540,215.1429,197.1314,187.1474,91.0569 |
| 70 | 16.27 | Phellodendron lactone | C26H30O8 | 470.19407 | [M+H]+ | 471.20134 | 471.20053 | -1.7 | 488460 | 471.3469,317.2180,161.0593 |
| 71 | 17.23 | Coptisinone | C19H13NO5 | 335.07937 | [M+H]+ | 336.08665 | 336.0871 | 1.3 | 8773 | 336.0850, 308.0885, 293.0663, 278.0779 |
| 72 | 17.25 | Anemoside D | C47H76O17 | 912.50825 | [M+H]+ | 913.51553 | 913.51507 | -0.5 | 23635 | 913.4993,751.4509,455.3522,437.3402,147.0715 |
| 73 | 17.71 | Diosgenin III | C36H58O9 | 634.40808 | [M-H]- | 633.40081 | 633.40076 | -0.1 | 3412 | 633.4152, 471.3462, 453.3356 |
| 74 | 17.72 | senkyunolide A | C12H16O2 | 192.11503 | [M+H]+ | 193.12231 | 193.1217 | -3.2 | 49272 | 175.1109,147.1172,137.0591 |
| 75 | 18.8 | 1H-2-Benzopyran-1-one,3,4-dihydro-6,8-dihydroxy-3-[2-(4-methoxyphenyl)ethyl]-, (3S)- | C18H18O5 | 314.11542 | [M-H]- | 313.10815 | 313.10816 | 0.0 | 175352 | 313.1063,298.0834,147.0443 |
| 76 | 18.96 | Anemoside A3 | C41H66O12 | 750.45543 | [M-H]- | 749.44815 | 749.4461 | -2.7 | 1303 | 749.4466,471.3562 |
| 77 | 22.98 | 23‐hydroxybetulinic acid | C30H48O4 | 472.35526 | [M-H]- | 471.34798 | 471.3472 | -1.7 | 115809 | 471.3468 |
| 78 | 23.52 | hederagenin | C30H48O4 | 472.35526 | [M-H]- | 471.34798 | 471.3472 | -1.7 | 115809 | 471.3471 |

**Supplementary figure**

The original images of the blots of the indicated proteins (The PVDF membranes were cut prior to developing the bands images).
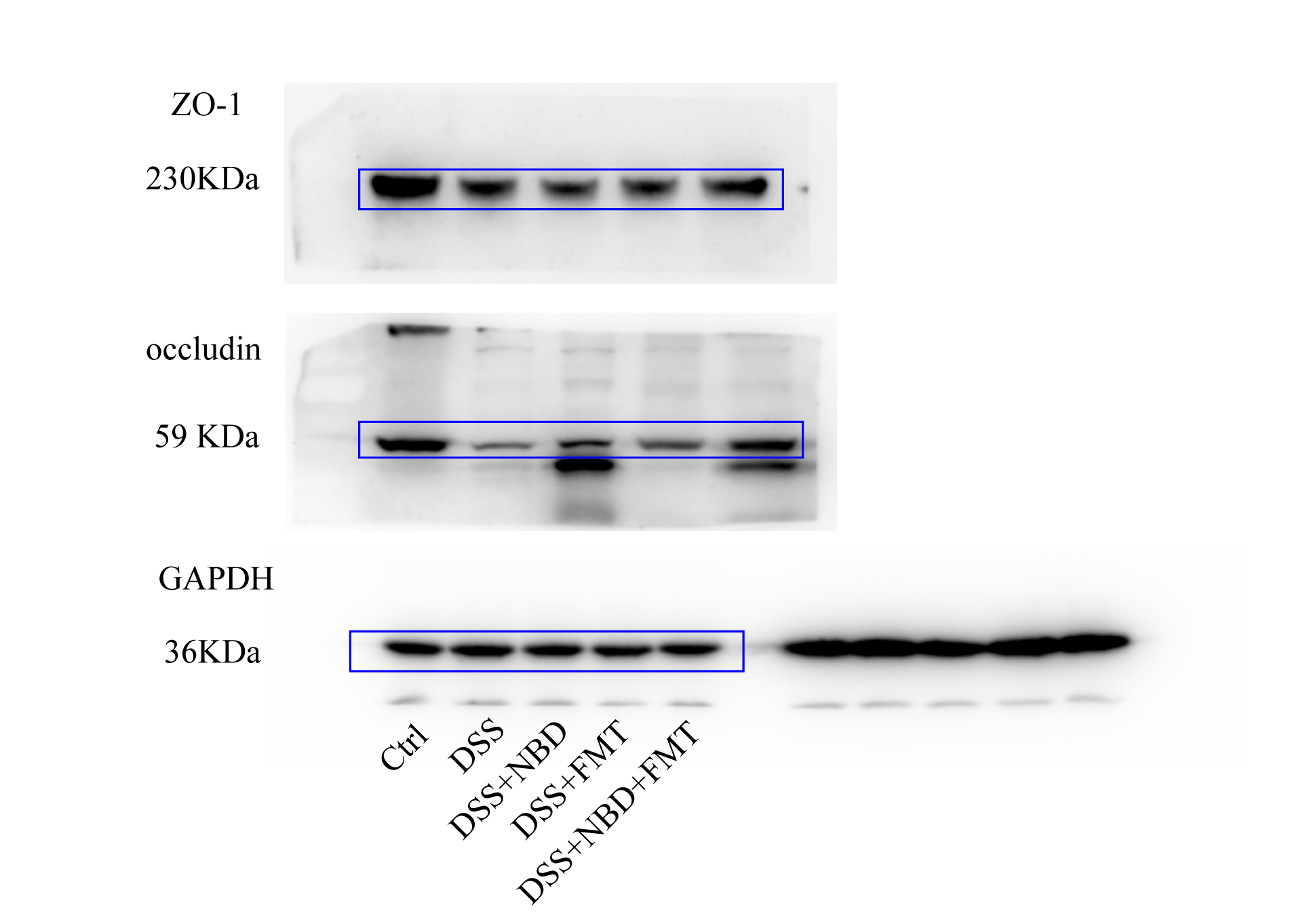


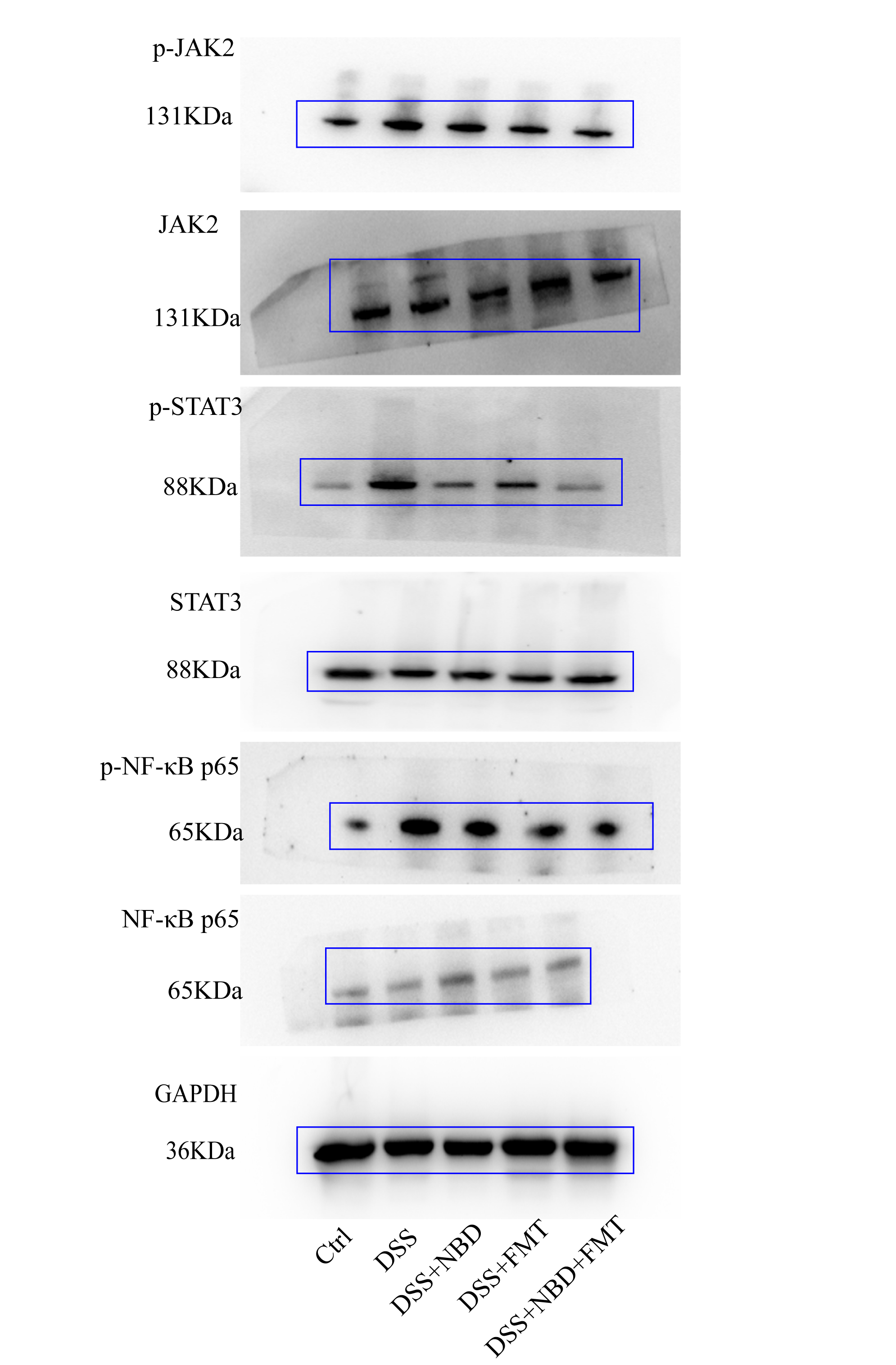

Supplement: Supplementary file 1 — Additional file 1. [file 12906_2022_3766_MOESM1_ESM.docx]
